# The Image Transceiver Device: Studies of Improved Physical Design

**DOI:** 10.3390/s8074350

**Published:** 2008-07-25

**Authors:** Yitzhak David, Uzi Efron

**Affiliations:** 1 Department of Electrical Engineering, Holon Institute of Technology, Holon, Israel; E-mail: isak@hit.ac.il; 2 Department of Electro-optical Engineering, Ben Gurion University, Beer-Sheva, Israel

**Keywords:** head-mounted display, CMOS imager, back illuminated APS, crosstalk, photo- activation, Smart-Goggle

## Abstract

The Image Transceiver Device (ITD) design is based on combining LCOS micro-display, image processing tools and back illuminated APS imager in single CMOS chip [1]. The device is under development for Head-Mounted Display applications in augmented and virtual reality systems. The main issues with the present design are a high crosstalk of the backside imager and the need to shield the pixel circuitry from the photo- charges generated in the silicon substrate. In this publication we present a modified, “deep p-well” ITD pixel design, which provides a significantly reduced crosstalk level, as well as an effective shielding of photo-charges for the pixel circuitry. The simulation performed using Silvaco software [ATLAS Silicon Device Simulator, Ray Trace and Light Absorption programs, Silvaco International, 1998] shows that the new approach provides high photo response and allows increasing the optimal thickness of the die over and above the 10-15 micrometers commonly used for back illuminated imaging devices, thereby improving its mechanical ruggedness following the thinning process and also providing a more efficient absorption of the long wavelength photons. The proposed deep p-well pixel structure is also a technology solution for the fabrication of high performance back illuminated CMOS image sensors.

## 1. Introduction

In several important Head Mounted Display (HMD) applications, there is a need to combine the Head Mounted Display capability with that of an imager. This stems from the need of superposing auxiliary information in real time, onto the field of view under observation. In order to accomplish this functionality, the scene must be imaged, processed and superposed self-aligned, and in real time, onto the observed field of view. Furthermore, from the standpoint of the imaging function we note that an Imager always requires a pointing or, an aiming function. Thus, an effective method of providing all of these functions is to have a device combining both functions of Imaging and Display, self-aligned with the observer's gaze. Adding to these requirements, the common HMD system needs for compactness and low-power consumption, it becomes clear that there is a significant advantage in combining both imaging and display functions, in a single chip. We have previously reported on the development of such a CMOS-based, integrated LCD/APS, Image Transceiver Device (ITD) chip for HMD applications [1].

**Figure 1.**
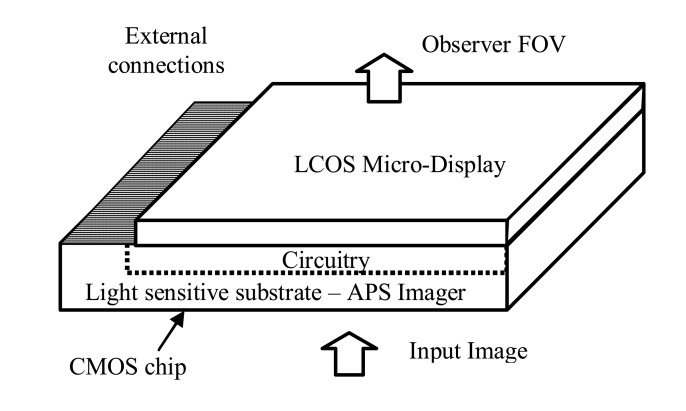
Basic ITD design.

The ITD design is based on combining LCOS micro-display and back illuminated APS imager structures on a common silicon substrate (fig. 1). The fabrication of this device using modern, high resolution CMOS process will result in the realization of a compact, head-mounted video system, comprising an imager, a micro-display and image processing tools [1, 2]. This ITD device is expected to find applications in head-mounted systems for augmented and virtual reality, as well as in Low – Vision Goggle serving as an aid for the visually impaired [3].

The realization of the imager and display pixel circuitry on a common silicon substrate while featuring a compact, efficiently combined Imager/Display structure, involves several issues, which cannot simply be overcome by common processing methods. The main issues are those of crosstalk and the need to shield the pixel circuitry from photo-charges generated in the silicon substrate.

One effective approach to suppress the pixel crosstalk in commonly used front side CMOS imager, is to prevent obliquely incident light from reaching the periphery of the photodiode. This is achieved by using one or more metal layers acting as photo-shields. Another way to reduce the crosstalk is to prevent the lateral diffusion of electrons to the adjacent pixel. In Particular, a deep p+ layer is used in [4], in order to block the diffusion of electrons generated below this layer towards the photodiode array. In [5], the low crosstalk level is achieved by double metal photo-shield as well as deep p-well imager structure. The photodiode array is formed in a low –doped p-well layer which provides a deeper photo conversion region.

However in a back- illuminated imager, neither approach is applicable. The crosstalk effect in back- illuminated photodiode array can be reduced by means of a guard-ring pixel electrode [6]. In that case, the array is realized on thin substrate chip (≤ 12 um), which is connected with a signal -processing chip, using flip-chip indium bonding technology.

In a reflective LCOS micro-display, the light-shielding problem of the LCD pixel switch transistor is solved by adding photo-shield metal layers to the existing light shielding mirror electrode matrix [7,8]. However, in the ITD structure the LCD switch transistor is also subject to the light flux incident on the backside imager. Consequently, the light-shielding problem requires a completely different solution.

In this work we present an ITD pixel structure design with a significantly reduced crosstalk level for the backside-configured imager, as well as an effective photo charge shielding for the pixel driving circuitry. In section 2 we present the crosstalk and circuitry shielding problems for the ITD structure, fabricated in a standard CMOS processes. In section 3 we introduce and analyze the “Deep p-well” ITD pixel structure as a potential solution, providing low crosstalk level for the backside-configured imager, as well as an effective photo charge shielding for the pixel driving circuitry. Studies of the behavior of the spectral response versus pixel thickness as well as the effect of surface recombination at the backside of the die on the ITD imager's spectral response, are also included in this section.

The analysis of the ITD pixel structures was performed using the Silvaco's ATLAS device simulator [9] with the Luminous module for simulating photo charge generation. The S-Pisces module was used for simulating charge transport and generation-recombination mechanisms by computing the Boltzman transport equations coupled with Shockley-Hall-Read, Auger and the optical generation-recombination models.

## 2. Basic Circuitry and Integrated Structures for the ITD Pixel

**Figure 2.**
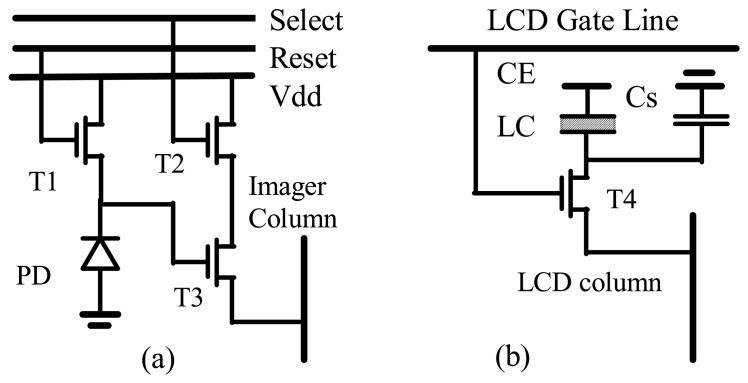
ITD pixel circuitry: a) – APS imager, b) LCD data switch. CE – common LCD electrode, LC – liquid crystal cell, C_S_ – storage capacitor, PD - photodiode.

The ITD pixel circuitry consists of an APS (Active Pixel Sensor) imager [10] and an LCD data switch circuitry [7, 8]. The APS circuitry (fig. 2, a) is a photodiode-based sensor with a reset switch (transistor T1), a select switch (transistor T2), a source follower (transistor T3) and an n-well / p- substrate diode.

The use of an APS in a front-illuminated imaging array configuration constitutes the basic circuitry of the commercially available CMOS imaging devices. The backside illuminated CMOS APS imager design provides an increased fill-factor, since the entire silicon substrate pixel area takes part in the light absorption process. However, backside illumination has worse spectral response and crosstalk properties.

The LCD circuitry (fig. 2, b) consists of a NMOS transistor switch (T4) and a storage capacitor connected to the LCD pixel electrode. It is also a typical data switch circuit of a LCOS (Liquid Crystal on Silicon) micro display.

**Figure 3.**
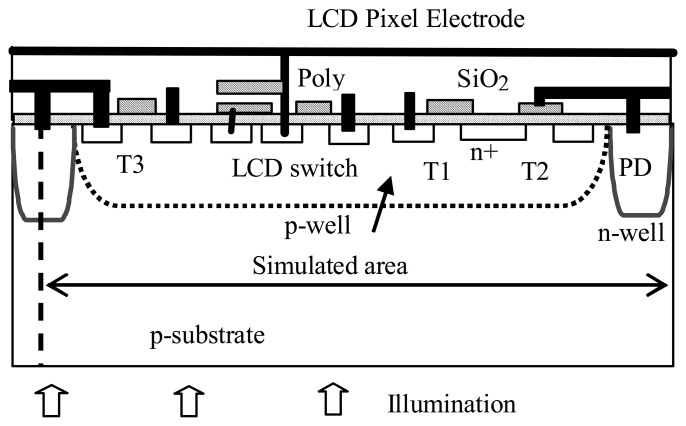
ITD structures: n-well and twin well (dotted line) pixels. The dotted line indicates the p-well boundary.

The basic ITD pixel structures realizing APS and LCD driving circuitry in the common silicon substrate are shown in fig. 3. These structures can be fabricated using standard, n-well and twin well CMOS processes. The sensor part of the structure consists of n-well/substrate photodiode and APS circuitry transistors (fig. 3, transistors T1, T2, T3). The transistor T4 (LCD switch) and the storage capacitor on the left-hand side of the structure are the components of the LCOS display circuitry, which is connected to the pixel display electrode. The transistors are formed in the p-substrate for n-well process based pixel and in the p-well region, (fig. 3, dotted line) for the twin-well-based pixel.

The imager part of the ITD operates in the back-side-illuminated configuration. Thus, the entire pixel area takes part in the absorption of the light impinging on the back surface of the die. The generated electrons spread in the substrate and can be collected by any p-n junction in the structure. This process degrades the performance of the imager in terms of crosstalk, photo response and leakage current, as well as the functioning of the LCD drive circuitry. The latter is due to the photocurrent generated at the switch transistor, by the stray, non-collected photoelectrons, which causes a discharge of the storage capacitor.

Thus, realization of these widely used circuits in the common pixel area of the silicon substrate requires the resolution of two problems: (a) maintaining a reasonable performance of the back illuminated imager; (b) light shielding of the LCD circuitry.

### 2.1. Simulation Aanalysis of the Standard CMOS Process Based ITD

**Figure 4.**
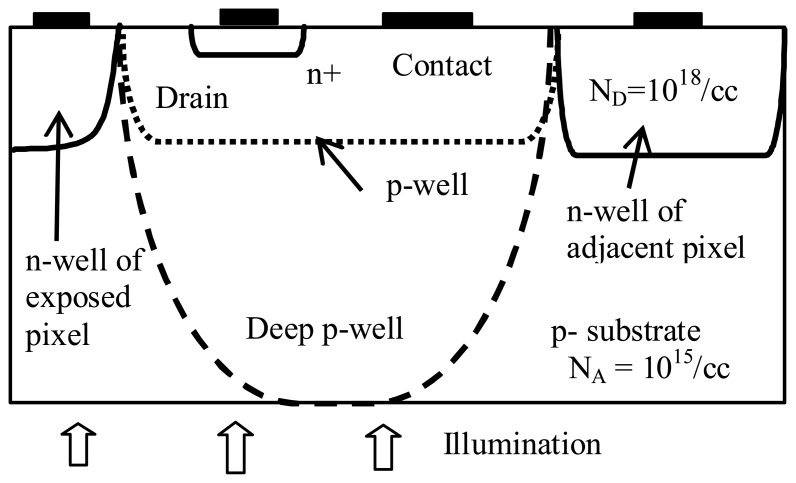
Simulated pixel structures: n-well (solid lines), twin well and deep p-well. The dotted line indicates the p-well boundary of the twin-well processed pixel. The dashed line indicates the boundary of the deep p-well region (See Section 3).

The simulated pixel structure for n-well process based ITD is shown in fig. 4. The n-well area on the left hand side of the structure is the photodiode of the illuminated pixel. The n-well area on the right hand side of the structure is the photodiode of the adjacent (non-illuminated) pixel. The n+ diffusion is the drain of the LCD pixel switch transistor (the “drain” region, in fig. 4).

In a twin-well process-based ITD structure, the pixel circuitry is placed inside the p-well area, doped at a level of 10^18^ /cc (fig. 4, the region enclosed by the dotted curve).

The 2-D steady-state analysis of these structures is performed under the following conditions: The n-wells and drain contact bias is 3V; the substrate contact (p-well contact for twin-well structure) is grounded, and the incident optical power density is 0.01 W/cm^2^.

**Figure 5.**
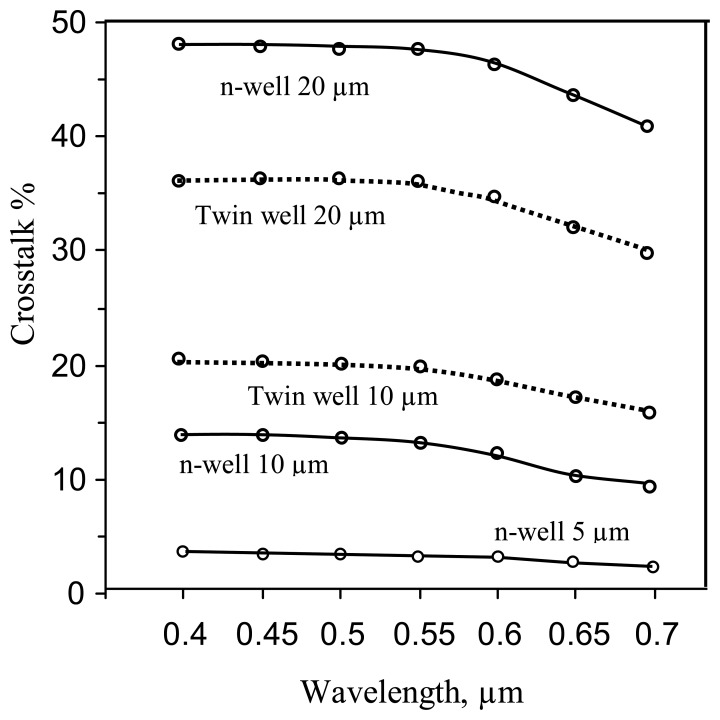
Crosstalk vs. optical wavelength (0.4 -0.7 μ) curves for various substrate thicknesses: solid curves – n-well pixel, dotted curves - twin well pixel.

The simulated results for the n-well process pixel structure shows that the crosstalk is 48% and 3% respectively, for the 20 μm and 5μm substrate thicknesses, at a wavelength of 0.55μm (fig.5, solid curves). The crosstalk is calculated as the ratio of the photo current of the adjacent, non-illuminated n- well diode, to the photo current of the exposed n-well diode. For the practically used thickness of 10μm, the crosstalk level is 15%, which is unacceptably high.

**Figure 6.**
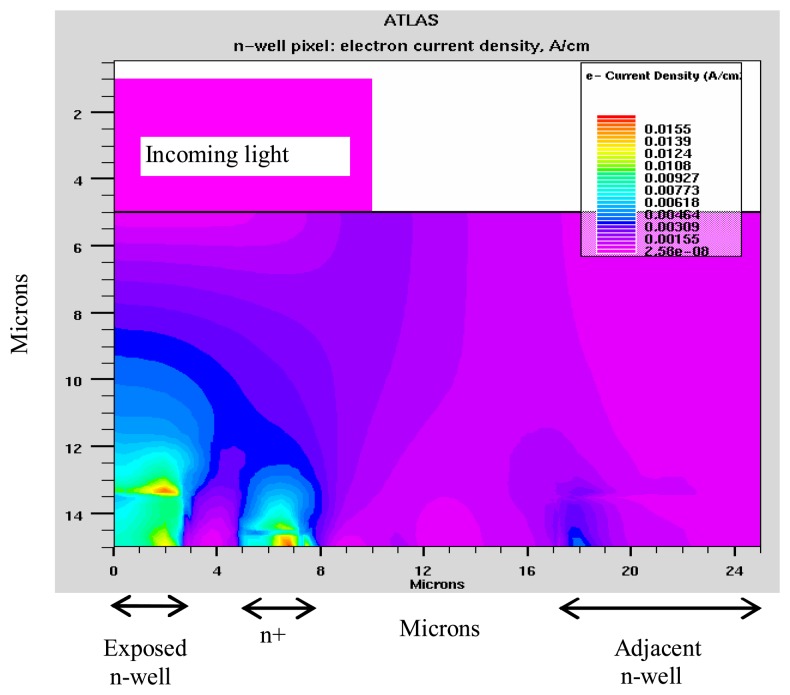
Electron current distribution for the n-well pixel.

These high crosstalk values are due to two processes. The first one is the lateral diffusion of the photoelectrons generated in the substrate into adjacent pixel, a process, which is typical in back illuminated imager [11]. The simulated results for the spreading photoelectrons current are shown in fig.6. The second, ITD-specific effect is the capture of photoelectrons by the n+/p-substrate “drain” diode, which is located in the exposed area of the pixel structure (n+ in fig.6). This diversion of photoelectrons away from the sensor photodiode and their capture by an LCD driving element, results in the following detrimental effects: (a) reduction of the effective quantum efficiency of the imager; (b) An effective increase in the crosstalk due to the reduction in the photocurrent of the exposed photodiode, and (c) Photo-activation of the drive circuitry which degrades its performance.

In particular, for a wavelength of 0.55μm and a die thickness of 10 μm, the photocurrent levels are as follows: Exposed pixel current = 0.55 pA; Adjacent pixel current = 0.075 pA; Drain diode current = 0.13 pA. It is seen, that the photoelectrons collected by the drain diode constitute a significant part of the exposed pixel photocurrent. These results, as pointed up above, in the reduction of the photo-diode current and therefore in the increase of the crosstalk, calculated as the ratio of the photo current of the adjacent n-well diode to the photo current of the exposed n-well diode.

The “drain”/substrate photocurrent grows with increasing wavelength and reaches levels as high as 10^-10^ A in the red part of the visible spectrum. This current discharges the LCD storage capacitor. The analysis of the equivalent lumped circuit of the LCD pixel circuitry shows that for a frame period of 30 ms, the storage capacitor voltage will drop by as much as three volts.

The general, wavelength-dependence trend of the simulated crosstalk and the photocurrents for the twin-well pixel structure do not significantly differ from those of the n-well structure, for short wavelengths (λ<0.7 μm). However, a dramatic reduction in the LCD transistor photocurrent is observed. The LCD transistor photocurrent of the twin-well pixel structure is almost two orders of magnitude lower than that of n-well pixel (table I).

This can be explained by the fact that the potential barrier at the p-well boundary blocks the diffusion of the generated photoelectrons into the p-well region thereby reducing the n+/p-well, drain diode photocurrent.

The simulated results also show that the photocurrent in both structures grows approximately linearly with increasing wavelength between 0.4 and 0.6 μm. In addition, the twin-well pixel photocurrent rises sharply at 0.7 μm. The linear dependence of the photo current in both structures is due to the increase in the number of generated electrons for the fixed incident optical power density (0.01 W/cm^2^) used. This dependence is of course quite universal and applies to all 3 structures studied. Now as previously explained, the effect of the p-well region is to divert the electrons generated outside the p-well region by short-wavelength photons (having sufficiently high absorption length), around the p-well area and into the n-well diode photo detector regions. However, for electrons generated within the p-well region there is no such blocking or diversion action of the p-well. Thus, these electrons, generated by long-wavelength photons, reach deep into the substrate and into the p-well region, due to their low absorption coefficient. These photo-generated electrons, with their long diffusion length of 40 micrometer, can now easily reach the transistor drain structure located within the p-well region (the n+ structure in fig. 3 above). The result is therefore a dramatic increase in the LCD transistor current with wavelength exceeding 0.6 micron, as is evidenced in Table 1. This effect is also clearly seen in the deep p-well case (see fig. 13 below).

As a consequence of decreasing of the n+/substrate diode photocurrent, a reduction of the crosstalk levels for relatively large values of die thickness (15-20 μm) is observed (fig.5). At the same time the n-well pixel shows a low crosstalk level for a thin 10μm die compared to that of a twin-well structure. This is due to the sinking action of the substrate contact, which in the case of a thin n-well structure, captures a certain fraction of the photo-generated carriers thus reducing the adjacent pixel current. Thus, in the thin n-well die, the substrate contact acts as a “guard ring electrode” to reduce the crosstalk. This action of the contact is weakened with increased die thickness, where the photoelectrons are able to flow “over” the contact area and reach the adjacent pixel, thereby increasing the cross-talk [6]. Now, in the case of the p-well structure, the photoelectrons generated outside the p-well region are diverted around the p-well region, as explained above, and are able to reach the adjacent pixels, with increasingly high cross-talk for thicker dice. So while the increase in the cross talk with increasing die thickness is common for both structures, the n-well has somewhat better performance (reduced cross talk) for very thin ∼10-μm dice.

However, the crosstalk level still remains unacceptably high. This agrees with previously reported results for a CMOS imager with a similar imager structure [12].

## 3. Deep P-well Pixel Structure

**Figure 7.**
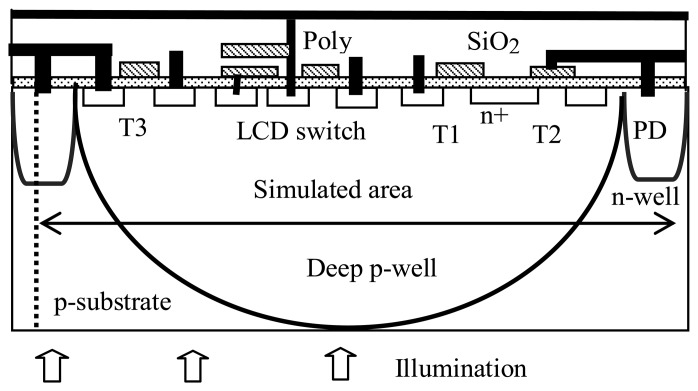
Physical structure of the “deep p-well” ITD pixel.

The Deep p-well structure is our proposed solution for the ITD pixel. This structure refers to the twin-well structure in which the p-well depth is equal to the substrate thickness (fig. 7 and fig. 4 with the region enclosed by the dash line).

**Figure 8.**
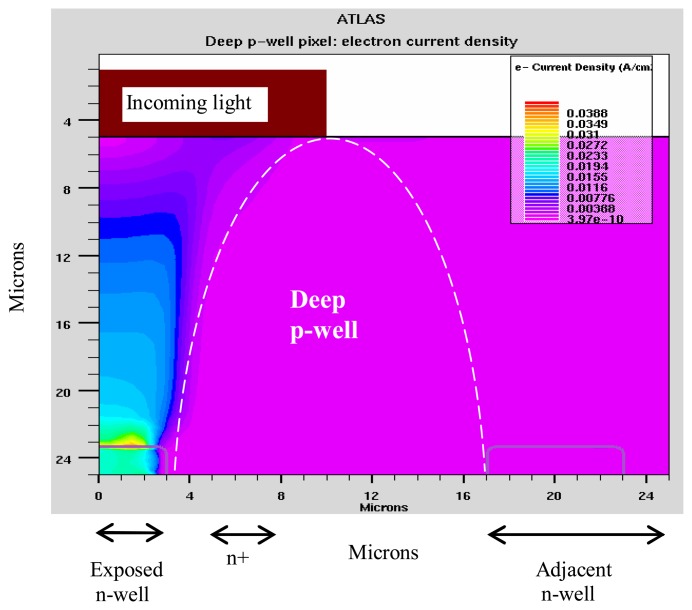
Electron current distribution for the deep p-well pixel.

The action of the deep p-well is based on the existence of a potential gradient from the p-well region to the other areas of the pixel. Thus, the electrons generated in the substrate and in the p-well region, are diverted to the photodiode region of the pixel, where they are collected. This is shown in fig. 8, which should be compared with the electron current distributions in the n-well structure case (fig.6).

**Figure 9.**
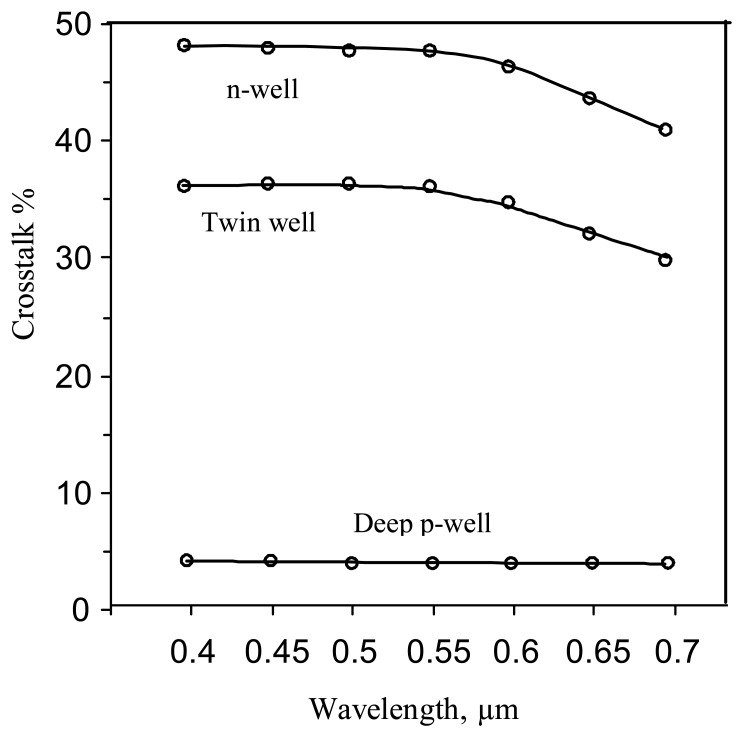
Cross-talk vs. optical wavelength (0.4 -0.7 μ) curves for deep p-well, n-well and twin-well structures; Die thickness =20 μm.

The simulated results show that the crosstalk level of this structure is much lower in comparison with that of the n-well and twin-well ITD and is now reduced to a few percent (fig. 9).

**Figure 10.**
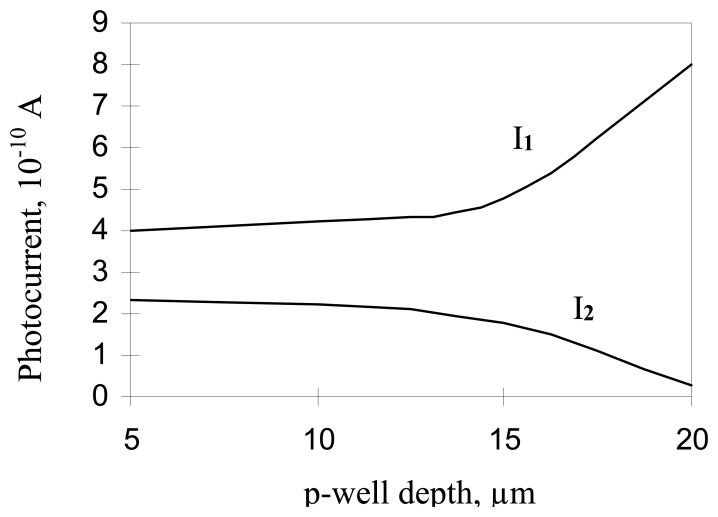
Photocurrents vs. p-well depth for die thickness of 20 μm, **at λ=0.55 μm**. I1 – exposed pixel current, I2 – adjacent pixel current.

The simulated photocurrents vs. p-well thickness, for a 20 μm die thickness, at λ=0.55 μm, are shown in fig. 10. It is seen, that maximum shielding effect takes place when the depth of the p-well is equal to the substrate thickness. In such a structure the p-well provides a potential gradient from the edges of pixel to its center, throughout the substrate thickness, up to the backside of the die. As previously explained, this potential gradient channels the photo generated electrons into the photodiode region. The simulated results show that even a few micrometer gap between the edge of the p-well and the backside edge of the die, is sufficient to generate a significant photoelectron leakage to the adjacent pixel, thereby increasing the crosstalk. This points to an important technology requirement namely, that the die should be thinned down to the edge of the p-well region.

It is also important to note, that this structure allows increasing the thickness of the die over the 10- 15 micrometers commonly used, thereby improving its mechanical ruggedness following the thinning process. It also provides a more efficient absorption of the long wavelength photons. The consideration of the ITD die thickness is addressed in the following section.

### 3.1 Trade-offs in the ITD Performance Parameters

There are three ITD performance parameters, which depend on the die thickness: The LCD switch transistor photocurrent, the crosstalk and photo-response. The general trends of these dependences with the increase in the die thickness are obvious: (a) the LCD photocurrent is expected to decrease to a level closer to the dark current; (b) the photo-response of the imager is expected to drop and, (c) the crosstalk is expected to increase. The question is – what is the maximum die thickness for acceptable levels of response and crosstalk?

The Silvaco s/w allows two options for simulating the imager response. The first one yields the ratio of the photodiode current to the equivalent current of the incident light (the source photo-current in Silvaco's terminology), a quantity which corresponds to the external quantum efficiency. The second is the ratio of the photodiode current to the equivalent current representing the highest possible rate of photons absorbed (The “Available current” in Silvaco's s/w terminology). This simulation option corresponds to simulating the internal quantum efficiency (IQE) of the device. The second option is the one we chose since we cannot take into account the reflection at the SiO2/Si interface.

**Figure 11.**
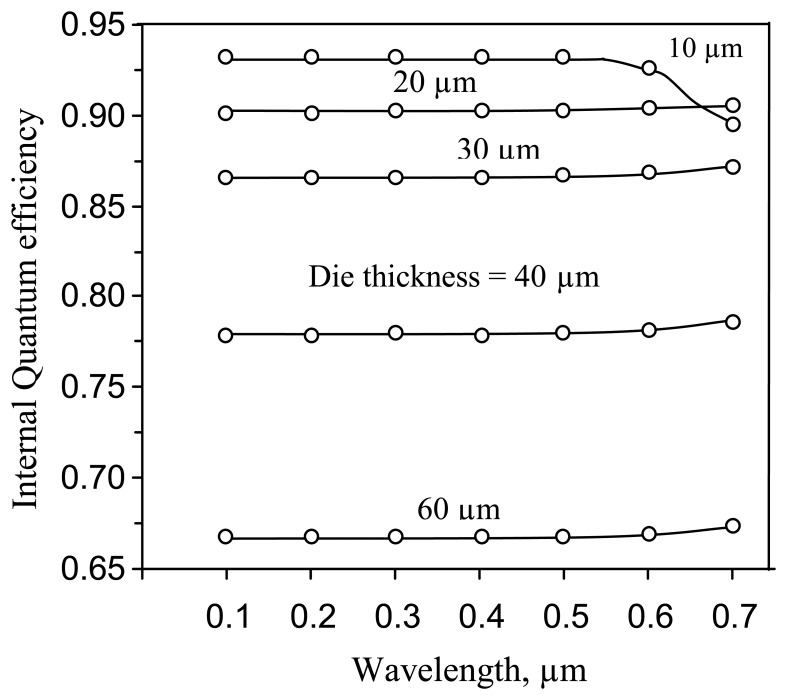
Imager IQE vs. optical wavelength for the deep p-well pixel.

The simulated IQE's spectral response (fig.11) shows the expected reduction in the imager quantum efficiency with the die thickness, where a thickness of 40 micrometer is comparable to the carrier diffusion length. Note, that the recombination model used, does not take into account the surface recombination on the backside of the substrate. Similar results for Quantum Efficiency were reported for a thinned, back illuminated CCD with backside accumulation and AR coating [17].

**Figure 12.**
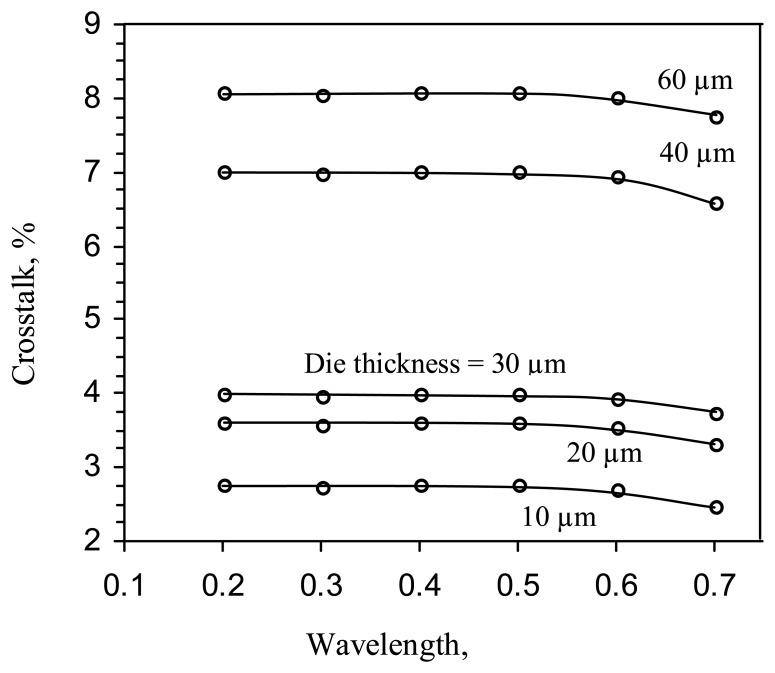
Crosstalk vs. optical wavelength for the deep p-well pixel.

The simulated crosstalk vs. the optical wavelength, with the die thickness as the variable parameter, is shown in fig.12. It is seen, that the crosstalk increases by almost two-fold, as the die thickness is increased from 30 to 40 μm.

**Figure 13.**
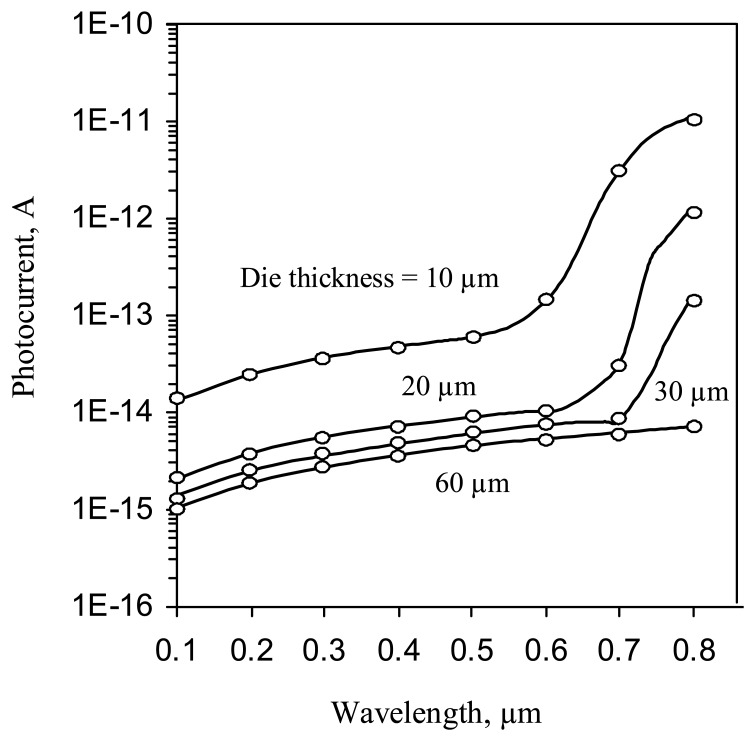
LCD transistor photocurrent for the deep p-well pixel.

At a thickness of 30 μm the LCD photocurrent (fig. 13) reaches a level of 0.1 pA for the red part of the visible spectrum. Note that this value of the LCD photocurrent is an order of magnitude lower than that of twin-well-based pixel (table 1). This level of photocurrent corresponds to a voltage drop of around 2 mV, obtained by approximating the LCD pixel voltage drop by: ΔV = I·T / C, where:

I - LCD photocurrent, T - frame period and C - LCD pixel capacitor, using typical values of: T = 20 msec and C = 1 pF, for the 0.1pA photocurrent level. This low level of voltage drop allows a 7-bit gray scale resolution, for ±2.5 volt data drive range, to be attained.

Thus, the corresponding die thickness of 30 um appears to be the best trade-off, as further reduction in the LCD photocurrent level, will necessitate further increase in the die thickness, which will result in the deterioration of the critical imaging parameters of photo-response and crosstalk, beyond the acceptable level. Table 2 summarizes the performance of the deep p-well pixel structure in comparison to standard -processed pixels, for a 30 μm die thickness.

Finally, it is interesting to note that the dramatic increase in the LCD transistor photocurrent for long wavelength (>0.7 μm) photons, discussed in section 2.1 above, is also clearly seen in this case of a deep p-well structure. Here, similar to the twin-well case, the p-well structure fails to divert the photo- electrons generated by the long wavelength photons, in the vicinity of the n+/p-well junction, within the p-well region. These electrons are therefore captured by the p-n junctions (n+/p-well) of the LCD transistor.

### 3.2 Surface Recombination at the Backside of the Die

Surface recombination in semiconductor devices is commonly described by a surface recombination velocity S_R_[12]. Silvaco s/w models the surface recombination using the bulk recombination model with effective carrier lifetime for the illuminated surface regions in the simulated structure. The effective carriers' lifetime calculation takes into account the surface recombination process [9].

The value of S_R_ at the backside of a silicon chip depends on the particular surface treatment. Surface recombination velocity can reaches 10^6^ – 10^7^ cm/sec for non-processed SiO_2_ / Si interfaces. A backside surface treatment after thinning and before AR deposition allows a significant reduction in the surface recombination and provides a high photo response.

Thus, as an example, the backside processing of the back illuminated CCDs required in order to attain high quantum efficiencies of 80-90% over the entire visible spectrum, involves backside surface passivation by charging, ion implantation or molecular beam epitaxy (MBE) growth of the thinned substrate, and the deposition of an anti-reflection coating [14,15]. Similar techniques for surface passivation are used for CMOS back illuminated image sensor [18].

**Figure 14.**
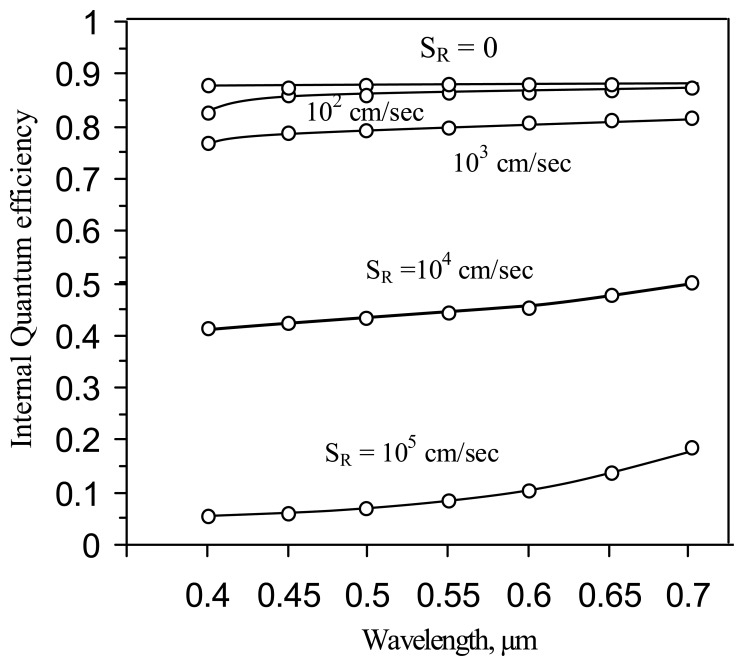
IQE response of the ITD imager with surface recombination: S_R_ - surface recombination velocity.

The simulated wavelength dependence of the IQE for the deep p-well ITD pixel, with a die thickness of 30 μm, in the presence of surface recombination at the back surface, is shown in fig. 14. The upper curve corresponds to a zero value of surface recombination velocity. In the case of low recombination velocities (S_R_ < 10^2^ cm/sec) the response of the ITD does not significantly differ from that of S_R_ = 0 (cm/s). Significant deterioration of the IQE response is observed for larger values of the recombination velocity (S_R_ > 10^3^ cm/sec). Similar dependences were reported in [16]. The conclusion is that the backside surface treatment commonly used in the back illuminated silicon devices technology will support the high quantum efficiency required for the ITD imager.

## 4. Conclusions

An improved design of a novel, CMOS based Image Transceiver Device is described.The device combines a front-side LCOS micro display with a back illuminated APS imager formed in a single- processed chip designed for Head Mounted Goggle application.

The main issues with the previous n-well-based, device design were a high imager crosstalk and a significant LCD transistor photocurrent (imager leakage current) level, resulting in an unacceptable fast discharge of the LCD storage capacitor. A solution of these issues by means of a “deep p-well” pixel structure is proposed. The proposed device design was studied using Silvaco's ATLAS device simulator. The simulation results show that the “deep p-well” configuration provides low crosstalk level, high photo response and a significantly reduced leakage photocurrent, required for a normal operation of the LCD part of the ITD. This configuration may therefore serve as the basis for the development of a high performance CMOS Image Transceiver Device. The proposed deep p-well pixel structure also constitutes a technology solution for the fabrication of high performance, back- illuminated CMOS image sensors.

## Figures and Tables

**Table 1.** LCD transistor photocurrent I_Dr_ for n-well and twin-well pixel structures vs. optical wavelength (substrate thickness = 20 um).

Wavelength, μm	0.4	0.5	0.6	0.7
n-well structure I_Dr_, pA	77	97	120	141
Twin-well structure I_Dr_, pA	0.071	0.089	0.144	1.84

**Table 2.** Simulation results for the three ITD pixel structures.

Sub. Thickness. 30 μm Wavelength: 0.55 μm	Cross Talk, %	LCD photocurrent, pA	Quantum Efficiency
Deep p-well	4	0.009	0.87
Twin-well	60	0.089	0.4
n-well	69	97	0.33

## References

[1] Efron U., David I., Sinelnikov V., Apter B. (2004). A CMOS/LCOS Image Transceiver Chip for Smart Goggle Application. IEEE Transaction on Circuits and System for Video Technology.

[2] Efron U., David I., Sinelnikov V., Friesem A.A. (2001). CMOS/LCOS-Based Image Transceiver Device. Spatial Light Modulators: Technology and Applications. Proceedings of SPIE.

[3] Efron U., David I., Apter B., Thirer N., Baal Zedaka I., Ben-Guigui A., Levy O., Nater P. (2005). A head-mounted, image transceiver-based, low vision aid.

[4] In Man Kang (2002). The Simulation of the Crosstalk between Photodiodes Fabricated Using 0.18 CMOS Process. School of Electrical Engineering, Seoul National University, SMDL Annual Report.

[5] Furumiya M. (2001). High Sensitivity and No-Crosstalk Pixel Technology for Embedded CMOS Image Sensor. IEEE Trans. Electron Devices.

[6] Hinckley S.P., Jans-Dravetzky V., Eshraghian K. (2004). Pixel Structure Effects on Crosstalk in backwall Illuminated CMOS Compatible Photodiode Arrays.

[7] Kurogane H., Doi K., Nishihata T., Furuya M., Nakagaki S., Takanashi I. (1998). Reflective AMLCD for Projection Display: D-ILA. SID Digest.

[8] Sanford J.L., Schlig E.S., Tomooka T., Enami K., Libsch F.R. (1998). Silicon Light-Valve Array Chip for High-Resolution Reflective Liquid Crystal Projection Displays. IBM J. Res. Develop..

[9] (1998). ATLAS framework integrated Silicon Device Simulator, Ray Trace and Light Absorption programs.

[10] Mendis S.K., Kemeny S.E., Gee R.C., Pain B., Staller C.O., Kim Q., Fossum E.R. (1997). CMOS active pixel image sensors for highly integrated imaging systems. IEEE J. Solid-State Circuits.

[11] Janesick J., Gunawan F., Dosluoglu T., Tower J., McCaffrey N. (2002). Scientific CMOS pixels. Experimental Astronomy, 14(1), Physics and Astronomy.

[12] Meynants G., Dierickx B., Scheffer D. CMOS active pixel image sensor with CCD Performance. Proc. of the SPIE/EUROPTO AFPAEC conference.

[13] Sze S.M. (2002). Semiconductor Devices, physics and technology.

[14] Burke B.E., Gregory J.A., Loomis A.H., Lesser M., Bautz M.W., Kissel S.E., Rathman D.D., Osgood R.M., Cooper M.J., Lind T.A., Ricker G.R. (2004). CCD Soft-X-Ray Detectors With Improved High- and Low-Energy Performance. IEEE Transactions on Nuclear Science.

[15] Huang C.M., Burke B.E., Kosicki B.B., Mountain R.W., Daniels P.J., Harrison D.C., Lincoln G.A., Usiak N., Kaplan M.A., Forte A.R.. (1989). A new process for thinned, back-illuminated CCD imager devices.

[16] Mackel H., Cuevas A. (2003). Determination of the surface recombination velocity of unpassivated silicon from spectral photoconductance measurements. Photovoltaic Energy Conversion, 2003. Proceedings of 3rd World Conference.

[17] Schaefer A.R., Varian R.H. (1990). Large area megapixel CCD arrays. Proceedings of the 33rd Midwest Symposium on Circuits and Systems.

[18] Pain B., Cunningham T., Nikzad S., Hoenk M., Jones T., Hancock B., Wrigley C. A back- illuminated megapixel CMOS image sensor. http://hdl.handle.net/2014/39312.

